# Impacts of cardiovascular risk factors and *APOE* on cognitive decline in late‐life

**DOI:** 10.1002/alz.092121

**Published:** 2025-01-09

**Authors:** Xinhui Wang, Isaac Asante, Lei Yu, Robert S. Wilson, David A. Bennett, Zoe Arvanitakis, Hussein N Yassine

**Affiliations:** ^1^ USC Keck School of Medicine, Los Angeles, CA USA; ^2^ Rush Alzheimer’s Disease Center, Rush University Medical Center, Chicago, IL USA

## Abstract

**Background:**

Apolipoprotein E (*APOE*) is a known genetic risk factor for dementia. Midlife cardiovascular risk factors are associated with lower cognitive performance and increased dementia risk. However, little is known whether cardiovascular risk factors mediate or modify the associations between *APOE* and cognitive decline in late‐life.

**Method:**

We conducted longitudinal data analyses on 1180 participants (aged 79.3±6.9 years at baseline) from Religious Orders Study and Rush Memory and Aging Project. Participants had ≥4 cognitive assessments with the last one within 2 years before death and *APOE* genotyped as ε4 carrier (ε3/ε4 or ε4/ε4) or non‐carrier (ε3/ε3). Annual global cognitive function was assessed via a battery of 19 tests and averaged into a summary measure. Cardiovascular risk factors included depression, hypercholesterolemia, and hypertension defined by corresponding medication usage in last 2 weeks at baseline, and diabetes based on self‐report or medication use. We constructed random change point models to analyze cognitive trajectories before death and estimate the subject‐specific change point, slopes before (pre‐terminal decline) and after (terminal decline) the change point, and level of cognition at death. We used linear regression to analyze the associations between *APOE*, cardiovascular risk factors, and these trajectory parameters adjusting for age at death, sex, and education. Interactions between *APOE* and risk factors were also examined.

**Result:**

On average, the onset of terminal decline happened at 2.9 years before death and the decline speed increased 5.6 times afterwards. Compared to non‐carriers, ε4 carriers had significantly lower cognition at death, earlier onset of terminal decline, faster pre‐terminal (β = ‐0.017, 95%CI = [‐0.023,‐0.012]) and terminal declines (β = ‐0.026, 95%CI = [‐0.044,‐0.008]) – equivalent to 2 years and 0.9 years of age effect respectively. Treated hypercholesterolemia and hypertension showed protective effects on cognitive trajectories, while depression showed adverse effect and no effect observed for diabetes. None of them changed the *APOE* adverse effects. No interactions were found between risk factors and *APOE* either (all p>0.05).

**Conclusion:**

*APOE* ε4 carriers were associated with worse global cognitive trajectories before death and these were not impacted by depression, hypercholesterolemia, hypertension, or diabetes. The protective effects of treated hypercholesterolemia and hypertension on cognitive trajectory did not differ by *APOE* genotype.

